# Objectively assessed sedentary time and type 2 diabetes mellitus: a case–control study

**DOI:** 10.1007/s00125-013-3051-5

**Published:** 2013-09-27

**Authors:** Mark Hamer, Sophie Bostock, Ruth Hackett, Andrew Steptoe

**Affiliations:** Department of Epidemiology and Public Health, University College London, 1–19 Torrington Place, London, WC1E 6BT UK

**Keywords:** Accelerometry, Case–control, Physical activity, Sedentary, Type 2 diabetes mellitus

*To the Editor:* There is some evidence to suggest detrimental, linear associations between objectively assessed sedentary time and various metabolic risk factors [1, 2], although it remains unclear if these associations are independent of moderate to vigorous physical activity [3, 4]. The effects of sedentary behaviour on health might be more apparent in clinical populations and the elderly, although the majority of research in this area has been conducted in healthy participants, which might partly explain inconsistencies in the findings. Thus, translation into specific clinical populations is needed. If a reduction in risk of type 2 diabetes mellitus can be achieved by rectifying the imbalance between sitting time and light-intensity (‘lifestyle’) activity, this would have important implications for early intervention and treatment. The aim of this study was to compare objectively assessed levels of sedentary and physical activity in type 2 diabetic patients and age matched healthy controls.

Healthy controls were drawn from a sub-sample of participants from the Whitehall II epidemiological cohort, as described previously [5]. Type 2 diabetic patients without history of cardiovascular disease were recruited from primary care clinics. Each diabetic patient was matched with two healthy controls based on age, sex and income. Participants gave full informed written consent to participate in the study and ethics approval was obtained from the University College London Hospital committee on the Ethics of Human Research.

Participants wore an accelerometer (Actigraph GT3X, Pensacola, FL, USA ) around the hip that records movement on the vertical and horizontal axis, during waking hours for 7 consecutive days. The accelerometer provides a measure of the frequency, intensity and duration of physical activity and allows classification of activity levels as sedentary, light, moderate and vigorous. The raw accelerometry data were processed using specialist software (MAHUffe, Cambridge, UK) to produce a series of standardised outcome variables. All participants included in the present analysis recorded a minimum of 10 h per day wear time and provided data for 5 days after exclusion of the first and last days of wear. Non-wear time was defined as intervals of at least 60 consecutive minutes of zero counts/minute (cpm). We used cut-off points previously described [5] to calculate daily times in each activity intensity band: sedentary (<1.5 metabolic equivalent of task [MET]): 0–199 cpm; light (1.5–3 MET) 200–1,998 cpm; moderate to vigorous physical activity (>3 MET): ≥1,999 cpm. All physical activity variables were converted to time (in minutes) per valid day. General linear models were employed to compare Actigraph data between cases and controls, making adjustments for wear time. All analyses were conducted using SPSS version 21 (IBM, Armonk, NY, USA).

Participants comprised 223 healthy controls (aged 64.0 ± 6.3 years; BMI 25.7 ± 3.8 kg/m^2^) and 122 type 2 diabetic patients (aged 63.9 ± 6.9 years; BMI 31.0 ± 5.6 kg/m^2^). In comparison with healthy controls, diabetic patients recorded more sedentary time (636 vs 662 min/day, *p* = 0.001), less light-intensity activity (208 vs 186 min/day, *p* = 0.02), but similar levels of moderate to vigorous activity (36 vs 33 min/day, *p* = 0.23) after adjusting for wear time. In further models that were adjusted for BMI (see Table 1), the differences in sedentary time were attenuated. Rates of smoking were higher among diabetic patients (13.8%) compared with healthy controls (5.4%), although further adjustment for smoking had negligible effects on the differences in sedentary time (14.8 min/day; 95% CI, −3.2, 32.7).

**Table 1:** The difference in sedentary time and physical activity between diabetic patients and healthy controls

Activity level	Model 1 *B* (95% CI)	Model 2 *B* (95% CI)
Sedentary time (min/day)		
Control group	Reference	Reference
Diabetic group	25.7 (10.3, 41.2)	15.0 (−2.6, 32.6)
Light activity (min/day)		
Control group	Reference	Reference
Diabetic group	−22.3 (−36.2, −8.4)	−17.7 (−33.5, −1.8)
Moderate to vigorous activity (min/day)		
Control group	Reference	Reference
Diabetic group	−3.3 (−8.8, 3.2)	2.9 (−3.3, 9.0)

Model 1 adjusted for wear time

Model 2 adjusted for wear time and BMI

The main findings from this study show differences in the balance between sedentary and light-intensity activity in diabetic patients and healthy controls. The greater time spent sedentary in diabetic patients was substantial (∼3 h per week) and might have aetiological relevance if this is reflective of habitual behaviour. The protective effect of physical activity becomes apparent with moderate-intensity exercise such as walking [6], although recent data in older adults has suggested that light activity also confers benefit in reducing the risk of type 2 diabetes [7]. The mechanisms may be linked to the displacement of sedentary time. A recent study showed that interrupting sitting time every 20 min with short 2 min bouts of light- or moderate-intensity walking lowered postprandial glucose and insulin levels in overweight/obese adults [8].

Given that the difference in sedentary time between diabetic patients and healthy controls was largely attenuated by adjustment for BMI, this suggests that excess weight might be a key barrier to activity. Indeed, a recent study in middle-aged adults demonstrated that BMI at baseline was prospectively associated with greater television viewing at follow-up, but not the converse [9].

The method used to assess sedentary time in this study has some limitations. The Actigraph quantifies time spent in different intensities of activity by summing time above and below specified count thresholds that may be less accurate for distinguishing sedentary and light activities. Thus, methods that employ postural allocation may be more reliable. The study was cross-sectional and we did not collect physical activity data prior to the development of diabetes. Nevertheless, physical activity behaviour appears to track from adulthood into older age [5]. Participants were matched for key variables although we were unable to control for other potentially important factors such as dietary intake and family history of diabetes.

In summary, type 2 diabetic patients recorded greater time in sedentary activity compared with healthy controls. Given the barriers to physical activity, it would be desirable if patients could gain benefit from incorporating relatively light levels of activity into their treatment modalities, which might be accomplished by simply reducing or breaking up sedentary time with movement. Patients may be more motivated by this approach, and becoming and staying active could seem more manageable.
